# Analysis of the *Mitogen-activated protein kinase kinase 4 *(*MAP2K4*) tumor suppressor gene in ovarian cancer

**DOI:** 10.1186/1471-2407-11-173

**Published:** 2011-05-17

**Authors:** Sally J Davis, David YH Choong, Manasa Ramakrishna, Georgina L Ryland, Ian G Campbell, Kylie L Gorringe

**Affiliations:** 1 VBCRC Cancer Genetics Laboratory, Peter MacCallum Cancer Centre, St. Andrew's Pl., East Melbourne, Victoria, Australia; 2 Department of Pathology, University of Melbourne, Parkville, Victoria, Australia; 3 Centre for Cancer Research, Monash Institute of Medical Research, Monash University, Clayton, Victoria, Australia

## Abstract

**Background:**

*MAP2K4 *is a putative tumor and metastasis suppressor gene frequently found to be deleted in various cancer types. We aimed to conduct a comprehensive analysis of this gene to assess its involvement in ovarian cancer.

**Methods:**

We screened for mutations in *MAP2K4 *using High Resolution Melt analysis of 149 primary ovarian tumors and methylation at the promoter using Methylation-Specific Single-Stranded Conformation Polymorphism analysis of 39 tumors. We also considered the clinical impact of changes in *MAP2K4 *using publicly available expression and copy number array data. Finally, we used siRNA to measure the effect of reducing *MAP2K4 *expression in cell lines.

**Results:**

In addition to 4 previously detected homozygous deletions, we identified a homozygous 16 bp truncating deletion and a heterozygous 4 bp deletion, each in one ovarian tumor. No promoter methylation was detected. The frequency of *MAP2K4 *homozygous inactivation was 5.6% overall, and 9.8% in high-grade serous cases. Hemizygous deletion of *MAP2K4 *was observed in 38% of samples. There were significant correlations of copy number and expression in three microarray data sets. There was a significant correlation between *MAP2K4 *expression and overall survival in one expression array data set, but this was not confirmed in an independent set. Treatment of JAM and HOSE6.3 cell lines with *MAP2K4 *siRNA showed some reduction in proliferation.

**Conclusions:**

*MAP2K4 *is targeted by genetic inactivation in ovarian cancer and restricted to high grade serous and endometrioid carcinomas in our cohort.

## Background

*Mitogen-activated protein kinase kinase 4 *(*MAP2K4) *is a gene encoding a member of the MAP kinase signalling family [1,2]. This 399 amino acid protein is a component of a triple kinase cascade whereby activated MAP kinases are successively phosphorylated to mediate cellular responses to cytokine signals, stress and other extracellular stimuli [3]. The phosphorylation cascade is initiated by the activation of MAP3K proteins, such as MEKK and MLK, which phosphorylate MAP2K4. In its activated state, MAP2K4 can phosphorylate JNK or p38 with dual specificity, resulting in the activation of the stress activated protein kinase (SAPK) pathway, which has been associated with apoptosis and neoplastic transformation [4,5]. However, the response initiated through the activation of JNK or p38 appears to be highly context dependent. For example, prolonged JNK activation has been demonstrated to induce an apoptotic effect in certain neuronal cells independent of c-Jun [6,7]. However, transient JNK activation in response to physiological stimuli does not elicit neuronal apoptosis, reflecting a dependence upon other factors to activate the JNK-mediated apoptotic pathway [8].

The genetic evidence for a role of *MAP2K4 *in cancer is gathering strength. It was initially described as a tumor suppressor gene (TSG), with a low frequency of deletions and mutations reported in a variety of cancer types [9]. In recent high-throughput sequencing and copy number studies, *MAP2K4 *appears to be only very rarely targeted by base pair level mutation but is consistently identified as a common target for deletion, including homozygous deletion [10-12]. While some of these deletions may be a consequence of LOH targeting the nearby *TP53 *gene on 17q12, in a few instances there are homozygous deletions that appear to target only *MAP2K4*. Despite this genetic support for a tumor suppressor role, functional studies have generated mixed results. One experimental model demonstrated that overexpression of *MAP2K4 *could reduce the incidence and onset of metastasis in ovarian and prostate cancer cell lines [13]. However, in contrast, overexpression and silencing of the gene in a different cellular context was found to support a pro-oncogenic role for *MAP2K4 *[14].

In ovarian cancer, in addition to the apparent role of *MAP2K4 *in metastasis suppression, there have been limited data showing loss of expression and genetic aberrations in several small cohorts [15-17]. Gene expression by immunohistochemistry and quantitative PCR was found to be reduced in cancer samples compared to normal or benign ovarian tissue. The only study to examine epigenetic control of *MAP2K4 *in any cancer type was performed on a small number of ovarian cancer cases, and did not find any evidence for promoter methylation [16]. We have previously reported that recurrent homozygous deletions appear to specifically target *MAP2K4 *in primary ovarian tumors, and the gene lay in a minimal region of loss of heterozygosity (LOH) in 68% (72/106) of the cases, the second most frequent locus targeted by LOH [18]. Despite this accumulating evidence, the status of *MAP2K4 *as a tumor suppressor in ovarian cancer is equivocal and therefore in this study we have undertaken a comprehensive analysis of mutation, methylation and gene knockdown.

## Methods

### Clinical specimens

One hundred and sixty one tumor tissue samples (77 serous, 21 mucinous, 33 endometrioid, 8 clear cell and 22 other, Additional file 1, Table S1) were obtained from patients presenting to hospitals in the south of England, UK or at the Peter MacCallum Cancer Centre, Australia. DNA was extracted either from whole tissue or from samples that were manually needle microdissected to ensure they contained >85% epithelial tumor cells. RNA was extracted from microdissection of subsequent 10 μm sections using the mirVana miRNA Isolation Kit (Ambion Inc, Austin, TX). Normal DNA was extracted from matching peripheral blood samples as described previously [19]. DNA for mutation screening underwent whole genome amplification (WGA) using the Repli-G Phi-mediated amplification system as described previously [20]. Institutional ethics committees approved this study.

### Mutation screening of MAP2K4 using High Resolution Melt (HRM)

Primers were designed to amplify each exon and the intron/exon boundaries of the *MAP2K4 *coding sequence using the software packages ExonPrimer and Primer3 [21], except for exon 11, which used previously published primers [12]. Exon 7 was covered by two overlapping primer pairs. Exon 1 was not analyzed as the PCR could not be optimized to amplify a specific product, despite multiple different primer sets, most likely because of the high GC content. Exon 1 contains 114 bp of coding sequence and has similarly been excluded from other mutation screening studies [12,22]. Primer sequences and amplification conditions are listed in Additional file 2, Table S2. HRM using PCR products amplified from WGA template DNA and DNA sequencing were carried out as described previously [20]. Somatic alterations were confirmed by re-sequencing from non-WGA tumor DNA and the matching normal DNA.

### MAP2K4 promoter methylation analysis by Methylation-Specific Single Strand Conformation Polymorphism (MS-SSCP) and bisulfite sequencing

The *MAP2K4 *CpG island was amplified using previously published oligonucleotides [16]. DNA samples were bisulphite treated using the MethylEasy Xceed Rapid DNA Bisulphite Modification kit (Human Genetic Signatures, Sydney, Australia) following the manufacturer's instructions. After PCR amplification, products were analyzed by SSCP using the ABI 3130 Genetic Analyzer (Applied Biosystems, Foster City, CA) as described previously [20], with *Sss*I methylase-treated normal DNA used as a positive control for CpG island methylation. Normal DNA samples were treated using CpG methyltransferase *Sss*I (New England Biolabs, Ipswich, MA), which methylates all CpGs before bisulfite treatment. Samples showing a shift in mobility were sequenced as described above.

### Knock-down of MAP2K4 in cell culture

Reagents for transient gene knockdown were obtained from Dharmacon (Thermo Fisher Scientific, Lafayette, CO), comprising the siRNA SMARTpools for *MAP2K4 *(L-003574-00) and a non-targeting control siRNA (D-001810-0X). This analysis was performed using the JAM and HOSE6.3 ovarian cell lines. The JAM cell line is a derivative of a poorly differentiated serous cystadenocarcinoma [23], while the HOSE6.3 cell line has been established by E6/E7 immortalisation of human ovarian surface epithelial cells [24].

Cells were cultured in 96-well plates at an initial density of 3.0 x 10^3 ^per well. Each cell line was reverse transfected [25]with 25 nmol/l of siRNA and the appropriate transfection reagent diluted in Optimem^® ^serum free medium (Invitrogen, Carlsbad, CA). HOSE 6.3 was treated with 0.05 μl DharmaFECT 1 and JAM was treated with 0.1 μl DharmaFECT 3. All transfections were performed in triplicate and in three separate experiments.

Transfection efficiency was determined by quantitative PCR (QPCR) whereby RNA was extracted from cells at 48 h post-transfection using the RNeasy Mini kit (Qiagen, Valencia, CA, USA), in accordance with the manufacturer's instructions. Thereafter, cDNA was generated from 100 ng of RNA using Superscript III VILO (Invitrogen, Carlsbad, CA), in accordance with the manufacturer's instructions. Expression of *MAP2K4 *was assessed using primers listed in Additional file 2, Table S2 on the LightCycler^® ^480 (Roche Diagnostics, Mannheim, Germany). PCR was performed using the SYBRgreen QPCR mix (Thermo Fisher Scientific, Lafayette, CO) and relative *MAP2K4 *gene expression was determined according to the comparative C_T _(ΔΔC_T_) method: ΔΔC_T _= ΔC_T _*MAP2K4 *siRNA DNA - ΔC_T _Non-targeting siRNA DNA. ΔC_T _(threshold cycles) is the C_T _of the reference gene minus the C_T _of the target gene. Fold difference of *MAP2K4 *expression was calculated by 2^-ΔΔCT^. *Hypoxanthine phosphoribosyltransferase 1 *(*HPRT1*) was used as an endogenously expressed reference gene for the purpose of quantifying relative gene expression. Each QPCR was performed in triplicate using cDNA derived from three independent experiments. Cellular proliferation was measured across a 7-day period according to the reduction of alamarBlue reagent (Invitrogen, Calsbad, CA) following a 4 h incubation as described previously [26].

### Statistical Analysis

Expression array files were imported into Partek Genomics Suite v 6.4 (Partek, St. Louis, MO) using default parameters including RMA normalization. These values were then used for statistical tests. Cox regression analysis was carried out in Partek Genomics Suite v 6.4. The results published here are in part based upon data generated by The Cancer Genome Atlas pilot project established by the NCI and NHGRI. Information about TCGA and the investigators and institutions who constitute the TCGA research network can be found at http://cancergenome.nih.gov. Other statistical analyses were carried out using GraphPad Prism software v5 (GraphPad Software, La Jolla, CA) and were considered significant at a P-value of less than 0.05. In assessing differences in proliferation, 2-way ANOVA was performed to interrogate time points as categorical variables.

## Results and Discussion

**Figure 1:**
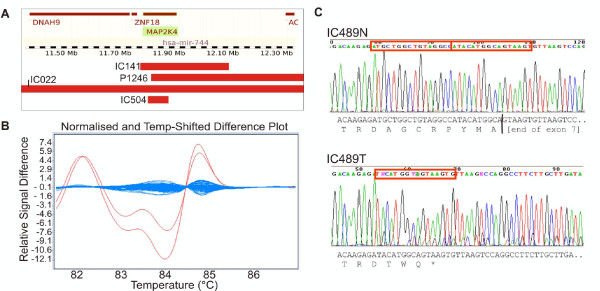
**Somatic alteration of *MAP2K4 *in ovarian cancer**. A. Homozygous deletions (red bars) over the *MAP2K4 *region detected by Affymetrix SNP array. B. An aberrant shift was detected by HRM (red trace) compared with the matching normal DNA and the other tumor samples (blue traces). C. The sequence trace of re-amplified, non-WGA tumor DNA (IC489T) compared to normal (IC489N) shows a 16 bp homozygous deletion in exon 7 (red box), which causes a frameshift alteration leading to premature termination of the protein as indicated by the sequence below.

We previously reported *MAP2K4 *as a candidate tumor suppressor gene [18] after SNP array copy number and LOH data from 125 primary ovarian tumors identified four homozygous deletions with a minimal point of overlap of just 50 kb, which targeted two genes, *MAP2K4 *and *hsa-mir-744*. In the current study we decided to undertake further analysis of these genes to assess the frequency of gene inactivation by means other than deletion (the analysis of *hsa-mir-744 *will be described elsewhere). We analyzed exons 2-11 of *MAP2K4 *in 149 primary ovarian cancers using HRM and DNA sequencing. We identified two somatic alterations, a homozygous 16 bp deletion in exon 7 in a serous ovarian tumor (Figure 1), and a heterozygous 4 bp deletion in exon 3 in an endometrioid tumor. The 16 bp deletion in IC489 causes a frameshift alteration that would lead to protein termination 7 amino acids downstream (p.Asp263fs). The mutation represented more than 70% of the bases in the sequence trace, thus we consider it to be homozygous, correlating with the SNP array data for this tumor showing chromosome 17 LOH across the *MAP2K4 *locus. The 4 bp deletion at the start of exon 3 would cause protein termination two amino acids downstream (p.Glu74fs) and is heterozygous in accordance with the lack of LOH at the locus in IC128 by SNP array.

No known or novel polymorphisms were detected in the coding region of *MAP2K4*, which is consistent with the low number (n = 5) of reported coding variants (dbSNP build 130, http://www.ncbi.nlm.nih.gov/projects/SNP/). Consequently, the failure to detect variants should not be attributed to a deficiency in the accuracy of the method, which has been successfully utilised to detect both novel and previously described polymorphisms in a similar context [20,27].

Combining the detection of mutation and deletion data, the overall frequency of homozygous inactivation of *MAP2K4 *was 5.6% (5/89 samples analysed by both techniques). However, it was notable that cases with genetic changes were either of the high-grade serous (4/5) or grade 3 endometrioid (1/5) subtypes. As a proportion of these subtypes, inactivation of *MAP2K4 *was 9.8% (4/41) and 4.8% (1/21), respectively. We also analyzed copy number data from The Cancer Genome Atlas (TCGA), a public data set of mostly high-grade serous ovarian tumors (http://cancergenome.nih.gov). In 157 SNP6.0 arrays, we identified a further 3 homozygous deletions, two of which specifically targeted only *MAP2K4*. TCGA has undertaken mutation screening by sequencing on 24 samples to date with no somatic alterations identified.

**Figure 2:**
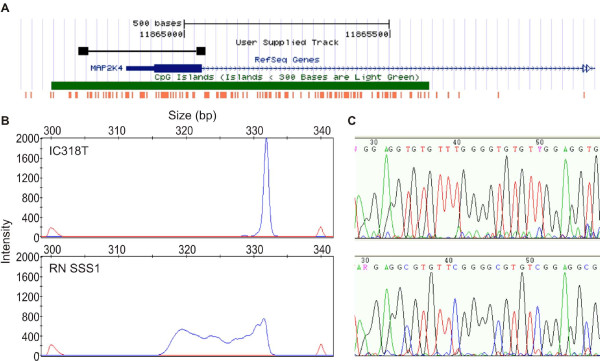
**Aberrant promoter hypermethylation is rare in the *MAP2K4 *promoter region**. A. Map of *MAP2K4 *promoter region from UCSC Genome browser (Mar. 2006 build) showing the gene in blue (exon 1 indicated by thicker bar), predicted CpG island in green, location of all CpG dinucleotides in orange, and location of the primers and MS-SSCP PCR product in black. MS-SSCP (B) and DNA sequencing electrophoretograms (C) for a representative tumor sample lacking methylation (top) and the positive methylation control *Sss*I treated normal lymphoblast DNA.

We had previously observed a decrease in *MAP2K4 *RNA expression in samples with LOH at the locus [18]. We analyzed promoter methylation by MS-SSCP and bisulfite sequencing to assess whether this reduction in expression was mediated by epigenetic mechanisms (Figure 2). We did not detect any electrophoretogram shifts in any tumor DNA that would indicate methylation in this region, in contrast to the positive *Sss*I treated control DNA. This was confirmed by selecting a few samples for sequencing. Consequently, methylation does not appear to be a mechanism by which *MAP2K4 *is silenced in ovarian cancer. In contrast, there was a high proportion of samples exhibiting LOH in conjunction with copy number loss at the *MAP2K4 *locus [18]. 56% of samples with LOH (38% of samples overall) were subject to copy number loss, indicating that *MAP2K4 *may be targeted by this genetic mechanism. In a recent copy number analysis [28] we found that 164/398 (41%) ovarian carcinomas of primarily serous/endometrioid subtype showed at least hemizygous copy number loss at *MAP2K4*. In addition, there was a strong correlation between copy number and gene expression in three microarray data sets of high grade serous/endometrioid ovarian carcinoma (Additional file 3, Figure S1): our own ([29]; r = 0.46, p = 0.001, n = 47), Etemadmoghadam *et al.*, ([30]; r = 0.59, p < 0.0001, n = 79), and TCGA (r = 0.51, p < 0.0001, n = 157). Therefore, the loss of *MAP2K4 *expression may be largely attributable to genomic loss, rather than epigenetic mechanisms of gene regulation.

To assess the impact of reduced *MAP2K4 *expression we used the full TCGA data (n = 400 with outcome data) and another published data set ([31], n = 207) to investigate any association with clinical outcome in invasive ovarian carcinoma (Additional file 4, Figure S2). Both data sets were comprised of primarily high-grade serous carcinomas. In a Cox regression analysis including age and residual disease as co-factors in the model, low expression of *MAP2K4 *was associated with improved overall survival in the Tothill data set (P = 0.031, hazards ratio = 1.7), but was not significant in the TCGA data set (P = 0.75). Expression of *MAP2K4 *was not significant in either data set when progression free survival was considered. When viewing this data using a Kaplan-Meier plot having split the samples based on quartiles of expression values, the difference in survival was associated only with the bottom quartile (low expressors) in the Tothill data set.

**Figure 3:**
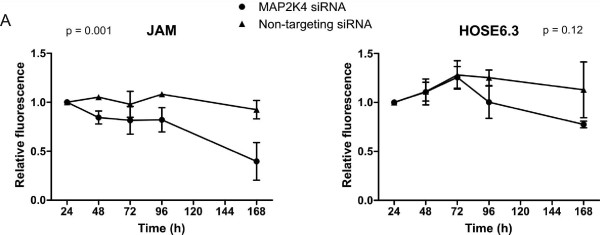
**Loss of *MAP2K4 *expression affects cellular proliferation**. Cellular proliferation was assessed using alamarBlue dye measured by fluorescence at 540/610 nm. Each of three experiments was performed in triplicate and normalized to the 24 h time-point. The average of three biological replicates is shown, except for the 168 h time-point, which was only done twice. Statistical significance was determined by 2-way ANOVA. Error bars are representative of SEM, n = 3.

To measure the effect of reduced *MAP2K4 *expression, we knocked down *MAP2K4 *in two cell lines using siRNA and assessed cellular proliferation (Figure 3). We used an immortalized cell line derived from human ovarian surface epithelium (HOSE6.3 [24]). While this line is not normal, it is non-tumorigenic and should have fewer disrupted pathways or additional genetic lesions than the cancer cell lines used previously. For comparison, we selected a cell line established in culture from an ovarian tumor xenograft, JAM [23], which expresses high levels of *MAP2K4*. Expression of *MAP2K4 *in transfected cells was evaluated at 48 h post-transfection by QPCR in each of the assays performed (Additional file 5, Figure S3). In each case, highly efficient gene silencing was observed, with at least 75% silencing of *MAP2K4 *expression in both cell lines analyzed. Cellular proliferation and metabolism was measured across a seven-day period. Both JAM and HOSE6.3 showed some reduction in cell number over this time course in the cells treated with *MAP2K4 *siRNA, however this was only significant for the JAM cells (P = 0.001, 2-way ANOVA). A recent study used a stable RNAi system to knock down expression of *MAP2K4*, and was able to select clones with reduced *MAP2K4 *expression, suggesting that loss of the gene may not always adversely affect cell viability or proliferation [32]. In this system the study found that *MAP2K4 *inactivation correlated with increased invasiveness through the induction of the epithelial to mesenchymal transition (EMT). Another recent study found that ectopic expression of *MAP2K4 *mutants increased anchorage-independent growth in NIH3T3 cells [33]. The effect of *MAP2K4 *gene knockdown may therefore be dependent on the *in vitro *experimental system used, perhaps not surprisingly given the role of the protein in such a complex signalling network.

## Conclusions

In summary, we have conducted a comprehensive analysis of mutation, methylation, expression and gene knockdown of *MAP2K4 *in ovarian cancer. We did not detect any methylation at the *MAP2K4 *promoter in ovarian tumour tissue, consistent with one other study to date [16]. We did find genetic alterations confirming *MAP2K4 *as a tumour suppressor gene for ovarian cancer in a subset of high grade serous and endometrioid cases, including a specific 16 bp homozygous deletion in exon 7 as well as larger homozygous deletions. In addition, we found that *MAP2K4 *was frequently targeted by hemizygous deletion and that this correlated with decrease in gene expression in several independent ovarian cancer data sets.

## Competing interests

The authors declare that they have no competing interests.

## Authors' contributions

IGC and KLG conceived of the study, participated in its design and coordination and helped to draft the manuscript. SJD performed the mutation, methylation and functional analyses and drafted the manuscript. DYHC and GLR assisted in the mutation and methylation analyses. MR assisted in the functional analyses. All authors read and approved the final manuscript.

## Pre-publication history

The pre-publication history for this paper can be accessed here:

http://www.biomedcentral.com/1471-2407/11/173/prepub

## Supplementary Material

Additional file 1 Table S1 Clinical samples analyzed in the mutation and methylation screens Click here for file

Additional file 2 Table S2 Oligonucleotide primer sequences Click here for file

Additional file 3 **Figure S1 Correlation of copy number with expression for *MAP2K4***. Three data sets are shown: Ramakrishna Gene1.0ST/SNP6.0 data [29], TCGA U133A/SNP6.0, and Etemadmoghadam U133Plus2/50K data [30]. For the U133 platforms probeset 203266_s_at is shown. Copy number is taken from the average copy number value of SNPs within the segment of lowest copy number intersecting with the *MAP2K4 *gene. R and p values for Pearson's correlation. Click here for file

Additional file 4 **Figure S2 Overall survival of ovarian cancer patients relative to *MAP2K4 *expression**. A. Partek output for Cox regression analysis in the Tothill data set of *MAP2K4 *expression probeset 203266_s_at, including patient age and residual disease as co-factors. B. Kaplan-Meier curves, showing difference in survival of patients with low *MAP2K4 *expression (bottom quartile of cases, red solid line) compared to all remaining cases (blue dashed line) from Tothill *et al.*, 2008 and The Cancer Genome Atlas (TCGA). Graphs truncated at 150 months. P value shown is the log rank (Mantel-Cox) test. Click here for file

Additional file 5 Figure S3 Reduction in *MAP2K4 *expression following siRNA knockdown Click here for file
