# Supplementary material for: Analysis of the Mitogen-activated protein kinase kinase 4 (MAP2K4) tumor suppressor gene in ovarian cancer
Source: BMC Cancer. 2011 May 17;11:173. doi: 10.1186/1471-2407-11-173 (PMC3115913; doi:10.1186/1471-2407-11-173)
Supplement: Additional file 1 — Table S1 Clinical samples analyzed in the mutation and methylation screens [file 1471-2407-11-173-S1.DOC]

**Supplementary Table 1 Mutation and methylation screen of ovarian cancer**

| Sample | Histological Subtype | Nature | Age | Grade | Stage | *MAP2K4* mutation | Exons screened | Methylation | Copy number/ Expression |
| --- | --- | --- | --- | --- | --- | --- | --- | --- | --- |
| IC019 | Serous | M | 86 | 3 | 3 |  | 3-11 | UM |  |
| IC022 | Serous | M | 65 | 3 | 3 | HD | 2-11 | UM | Y/Y |
| IC023 | Serous | M | 47 | 1 | 1 |  | NS | UM |  |
| IC026 | Serous | M | 51 | 2 | 3 |  | 2-11 |  | Y/Y |
| IC027 | Serous | M | 47 | 2 | 1b |  | 2-11 |  |  |
| IC030 | Endometrioid/ Mucinous | M | 59 | 3 | 3 |  | 2-11 |  |  |
| IC032 | Serous | M | 71 | 1 | 2 |  | 2-11 |  | Y/N |
| IC036 | Endometrioid | M | 54 | 1 | 1a |  | 2-11 |  |  |
| IC040 | Mucinous | M | 73 | 3 | 2 |  | 2-11 |  | Y/N |
| IC045 | Serous | M | 50 | 3 | 3 |  | 2-11 |  |  |
| IC047 | Endometrioid | M | 50 | 3 | 2 |  | 2-11 |  |  |
| IC048 | Serous | M | 50 | 2 | 3 |  | 3-11 |  |  |
| IC049 | Mullerian | M | 49 | 3 | 3 |  | 2-11 |  | Y/N |
| IC050 | Mucinous | M | 81 | 2 | 1 |  | 2-11 | UM | Y/N |
| IC060 | Granulosa Cell Tumour | M | 47 | 1 | 1a |  | 2-11 |  |  |
| IC061 | Mucinous | M | 76 | 1 | 1a |  | 2-11 |  |  |
| IC063 | Serous | M | 66 | 3 | 1 |  | 2-11 |  |  |
| IC070 | Endometrioid | M | 57 | 1 | 1a |  | 2-11 | UM |  |
| IC071 | Serous | M | 68 | 2 | 1a |  | 3-10 | UM |  |
| IC075 | Mucinous | B | 68 | 0 | - |  | 2-11 |  |  |
| IC078 | Endometrioid | M | 76 | 2 | 1a |  | 2-11 |  |  |
| IC080 | Mucinous | M | 71 | 1 | 1a |  | 2-11 | UM | Y/N |
| IC086 | Serous | M | 84 | 3 | 3a |  | 2-11 |  | Y/N |
| IC095 | Endometrioid | M | 66 | 3 | 2 |  | 2-11 |  | Y/Y |
| IC114 | Endometrioid | M | 70 | 2 | 1a |  | 2-11 |  |  |
| IC119 | Serous | M | 59 | 2 | 1c |  | 2-11 |  |  |
| IC121 | Mucinous | M | 77 | 1 | 3 |  | 2-11 |  | Y/N |
| IC122 | Serous | M | - | 3 | 3 |  | 2-11 | UM |  |
| IC124 | Granulosa Cell Tumour | M | 53 | - | 3 |  | 2-11 | UM |  |
| IC128 | Endometrioid | M | 56 | 2 | 1c | 4 bp del (het)  (p.Glu74fs) | 2-11 |  | Y/N |
| IC131 | Serous | M | 74 | 3 | 3 |  | 2-11 |  | Y/N |
| IC134 | Serous | M | 64 | 3 | 3c |  | 2-11 | UM |  |
| IC135 | Serous | M | 66 | 2 | 2 |  | 2-11 |  | Y/N |
| IC136 | Endometrioid/ Clear Cell | M | 78 | 3 | 1 |  | 2-11 |  |  |
| IC138 | Mucinous | M | 47 | 1 | 1a |  | 2-11 | UM | Y/N |
| IC139 | Serous | M | 65 | 3 | 3 |  | 2-11 | UM |  |
| IC141 | Serous | M | 56 | 3 | 3c | HD | 2-11 |  | Y/N |
| IC142 | Serous | M | 66 | 3 | - |  | 2-11 |  |  |
| IC144 | Mucinous*/ Endometrioid | M | 63 | 1 | 1c |  | 2-11 |  |  |
| IC146 | Endometrioid | M | 67 | 2 | 1c |  | 2-11 | UM |  |
| IC149 | Serous | B | 66 | 0 | 0 |  | 2-11 | UM |  |
| IC151 | Endometrioid | M | 56 | 2 | 2c |  | 2-11 |  | Y/N |
| IC154 | Serous | M | 52 | 2 | 3a |  | 2-11 |  |  |
| IC156 | Mucinous | B | 47 | 0 | - |  | 2-11 |  |  |
| IC170 | Endometrioid | M | 60 | 2 | 1a |  | 2-11 |  |  |
| IC176 | Mucinous | M | 62 | 1 | 1a |  | 2-11 |  |  |
| IC177 | Endometrioid | M | 53 | 3 | 1a |  | 2-11 |  |  |
| IC179 | Endometrioid | M | 62 | 3 | 3 |  | 2-11 | UM | Y/Y |
| IC184 | Adenocarcinoma | M | 47 | 3 | 2 |  | 2-11 |  |  |
| IC186 | Mucinous | BL | 78 | 0 | 1c |  | 2-11 |  |  |
| IC194 | Papillary | M | 63 | 3 | 2 |  | 3-11 |  |  |
| IC197 | Mixed Mullerian Tumour | M | 50 | 3 | 3 |  | 2-11 |  |  |
| IC201 | Serous | M | 80 | 2 | 3b |  | 2-11 | UM | Y/Y |
| IC202 | Endometrioid | M | 65 | 1 | 2 |  | 2-11 |  |  |
| IC204 | Serous | M | 73 | 3 | 3c |  | 2-11 |  |  |
| IC215 | Serous | M | 55 | 3 | 1c |  | 2-11 |  |  |
| IC219 | Mucinous | M | 63 | 1 | 1c |  | 2-11 |  | Y/N |
| IC220 | Endometrioid | M | 62 | 2 | 3c |  | 2-11 |  | Y/N |
| IC252 | Endometriosis | B | - | 0 | 0 |  | 2-11 |  |  |
| IC253 | Endometrioid | M | 70 | 2 | 3a |  | 2-11 |  |  |
| IC257 | Mucinous | M | 60 | 1 | 1a |  | 2-9, 11 |  | Y/N |
| IC258 | Endometrioid | M | 69 | 3 | 3c |  | 2-11 | UM | Y/Y |
| IC263 | Mucinous | BL | 62 | 0 | 1a |  | 2-11 |  |  |
| IC277 | Serous | M | 71 | 3 | 3c |  | 2-11 |  | Y/N |
| IC281 | Serous | M | 80 | 2 | 1b |  | NS |  | Y/N |
| IC282 | Serous | M | 73 | 3 | 3 |  | 2-11 |  |  |
| IC285 | Serous | M | 61 | 2 | 2c |  | 2-11 |  |  |
| IC286 | Endometrioid | M | 71 | 3 | 3b |  | 2-11 |  | Y/N |
| IC288 | Serous | M | 51 | 2 | 3 |  | 2-11 |  | Y/Y |
| IC291 | Serous | M | 87 | 3 | 3 |  | 2-11 |  |  |
| IC292 | Mucinous | M | 80 | 2 | 3 |  | 2-11 |  | Y/N |
| IC293 | Endometrioid | M | 59 | 3 | 1 |  | 2-11 |  | Y/Y |
| IC297 | Serous | M | 53 | 3 | 3 |  | 2-11 |  |  |
| IC300 | Endometrioid | M | 68 | 3 | 3 |  | 2-11 | UM | Y/Y |
| IC302 | Serous | M | 64 | 2 | - |  | 2-11 |  |  |
| IC307 | Endometrioid | M | 57 | 3 | - |  | 2-11 |  | Y/N |
| IC314 | Serous | M | 74 | 3 | - |  | 2-11 |  |  |
| IC315 | Serous | M | 82 | 3 | - |  | NS | UM | Y/Y |
| IC318 | Serous | M | 70 | 3 | 3 |  | 2-5, 7-11 | UM | Y/Y |
| IC321 | Mucinous | M | 47 | 2 | - |  | 2-11 |  | Y/N |
| IC323 | Serous | M | 54 | 2 | - |  | 2-11 | UM | Y/N |
| IC325 | Serous | M | 64 | 3 | - |  | NS |  | Y/Y |
| IC328 | Serous | M | 66 | 2 | - |  | 2-11 | UM | Y/Y |
| IC342 | Mixed Mullerian Tumour | M | 54 | 0 | - |  | 2-11 |  |  |
| IC343 | Mucinous | M | 53 | 1 | - |  | 2-11 |  | Y/N |
| IC349 | Clear Cell | M | 39 | 2 | - |  | 2-11 |  | Y/N |
| IC356 | Granulosa Cell Tumour | M | 64 | 0 | 1 |  | 2-11 |  |  |
| IC359 | Endometrioid | M | 57 | 2 | 1a |  | 2-11 |  | Y/N |
| IC363 | Adenocarcinoma | M | 54 | 3 | 3+ |  | 3-7, 9-11 |  |  |
| IC365 | Serous | M | 58 | 2 | - |  | 2-11 |  |  |
| IC374 | Endometrioid | M | 80 | 2 | 3 |  | NS |  | Y/N |
| IC375 | Endometrioid | M | 83 | 3 | - |  | NS |  | Y/N |
| IC380 | Adenocarcinoma | M | 64 | 3 | 3 |  | 3-11 |  |  |
| IC381 | Mucinous | M | 73 | 3 | 1c |  | NS |  | Y/N |
| IC382 | Serous | M | 59 | 3 | 3 |  | 2-11 |  | Y/Y |
| IC399 | Endometrioid | M | 66 | 3 | 4 |  | 2-11 |  | Y/N |
| IC400 | Mucinous | M | 66 | 1 | 3 |  | 2-11 |  | Y/N |
| IC403 | Mucinous | M | 64 | 3 | 3 |  | NS |  | Y/N |
| IC406 | Serous | M | 76 | 3 | - |  | NS |  | Y/N |
| IC407 | Serous | M | 83 | 2 | 3 |  | 2-11 |  | Y/Y |
| IC410 | Endometrioid | M | 51 | 2 | 3 |  | NS |  | Y/N |
| IC413 | Serous | M | 36 | 3 | 3 |  | NS |  | Y/Y |
| IC419 | Clear Cell | M | 65 | 1 | 1 |  | 2-11 |  | Y/N |
| IC425 | Fibroma | B | 49 | 0 | 0 |  | 2-11 |  |  |
| IC432 | Endometrioid | M | 78 | 1 | 1c |  | 3-11 | UM |  |
| IC434 | Endometrioid | M | 76 | 3 | 1c |  | 2-11 |  | Y/Y |
| IC435 | Mucinous | M | 69 | 2 | 1 |  | NS |  | Y/N |
| IC445 | Endometrioid | M | 62 | 1 | 1a |  | NS |  | Y/N |
| IC438 | Serous | B | 57 | 0 | - |  | 2-11 |  |  |
| IC448 | Mucinous | M | 55 | 1 | 1c |  | 2-11 |  | Y/N |
| IC450 | Serous | B | 68 | 0 | - |  | 2-3, 5-11 |  |  |
| IC474 | Serous | M | 56 | 2 | 1c |  | 2-11 |  | Y/N |
| IC476 | Endometrioid | M | 73 | 1 | 1c |  | 2-11 |  |  |
| IC477 | Serous | M | 71 | 3 | 3 |  | NS |  | Y/N |
| IC478 | Endometrioid | M | 71 | 2 | 1a |  | 2-3, 5-11 |  | Y/N |
| IC487 | Serous | M | 41 | 1 | 3 |  | NS |  | Y/N |
| IC489 | Serous | M | 42 | 3 | 3 | 16 bp del (hom)  (p.Asp263fs) | 2-8, 10-11 |  | Y/N |
| IC490 | Mucinous | M | 49 | 2 | 1 |  | 2-11 |  | Y/N |
| IC491 | Serous | M | 52 | 3 | 4 |  | 2-3, 5-11 |  |  |
| IC493 | Serous | M | 61 | 2 | 3 |  | NS | UM | Y/Y |
| IC498 | Serous | M | 80 | 2 | 3 |  | 2-11 |  |  |
| IC499 | Serous | M | 49 | - | 2 |  | NS | UM | Y/Y |
| IC504 | Endometrioid | M | 50 | 3 | 1 | HD | NS |  | Y/Y |
| IC509 | Serous | M | 66 | 2 | 3 |  | NS | UM | Y/N |
| IC511 | Clear Cell | M | 61 | 1 | 3 |  | NS |  | Y/N |
| IC519 | Serous | M | 64 | 2 | 1c |  | NS |  | Y/N |
| IC526 | Endometrioid | M | 74 | 2 | 3 |  | NS |  | Y/N |
| IC533 | Endometrioid | M | 87 | 3 | 4 |  | 2-11 |  | Y/N |
| IC548 | Serous | M | 61 | 1 | 3 |  | 2-11 |  | Y/N |
| IC549 | Clear Cell | M | 42 | 3 | - |  | NS | UM | Y/N |
| IC551 | Serous | M | 55 | 3 | 3 |  | NS |  | Y/N |
| IC557 | Mucinous | M | 35 | 1 | 1 |  | 2-11 |  | Y/N |
| IC559 | Brenner Tumour | M | 72 | 1 | - |  | 2-11 |  |  |
| IC565 | Serous | M | 63 | 2 | 2 |  | 2-11 |  | Y/N |
| IC569 | Serous*/ Endometrioid | M | 71 | 3 | 3c |  | 2-11 |  |  |
| IC579 | Serous | M | 71 | 3 | - |  | NS |  | Y/Y |
| IC580 | Endometrioid | M | 70 | 2 | 1c |  | 2-11 |  | Y/N |
| IC594 | Endometrioid | M | 47 | 3 | 1 |  | NS | UM | Y/Y |
| P1010 |  |  |  |  |  |  | NS | UM |  |
| P0505 | Endometrioid | M | 59 | 3 | 2 |  | 2-11 |  | Y/N |
| P0511 | Clear cell | M | 69 | 3 | 2b |  | 2-11 |  | Y/N |
| P0532 | Serous | M |  |  |  |  | 2-11 |  |  |
| P0566 | Mixed (Serous*) | M | 71 | 3 | 3b |  | 2-11 |  | Y/N |
| P0667 | Serous | M | 56 | 3 | 2b |  | NS |  | Y/N |
| P0706 | Serous | M | 57 | 2 | 3c |  | NS |  | Y/Y |
| P0756 | Serous | M | 66 | 3 | 2 |  | NS |  | Y/N |
| P0772 | Serous | M | 36 | 3 | 2c |  | NS |  | Y/N |
| P0933 | Serous | M | 85 | 3 | 2c |  | 2-11 |  | Y/Y |
| P0985 | Adenocarcinoma | M | 88 | 3 | 2c |  | 2-11 |  | Y/Y |
| P1005 | Serous | M | 65 | 3 | 3c |  | NS | UM |  |
| P1049 | Serous | M | 50 | 3 | 2 |  | 2-11 |  | Y/Y |
| P1094 | Clear Cell | M | 56 | - | 1a |  | 2-11 |  | Y/N |
| P1246 | Serous | M | 69 | 2 | 2c | HD | 2-11 |  | Y/Y |
| P1348 | Serous | M | 41 | 3 | 2 |  | 2-11 |  | Y/Y |
| P1389 | Mixed Mullerian Tumour | M | 67 | 3 | 2c |  | NS | UM | Y/N |
| P1428 | Serous | M | - | 2 | 2a |  | 2-11 |  | Y/Y |
| P1436 | Serous | M | 61 | 3 | 3c |  | NS |  | Y/Y |
| P1555 | Serous | M | 52 | 3 | 2b |  | NS | UM | Y/Y |
| P1556 | Mixed (Clear Cell/Serous*) | M | 39 | 3 | - |  | 2-11 |  | Y/N |
| P1680 | Clear Cell | M | 70 | 3 | 3 |  | 2-11 |  | Y/N |
| P1705 | Serous | M | 81 | 3 | 2 |  | 2-11 |  | Y/N |
| P1768 | Endometrioid | M | 51 | 2 | 1a |  | 2-11 | UM | Y/N |
| P1805 | Serous | M | 64 | 2 | 3b |  | 2-11 | UM | Y/N |
| P1854 | Serous | M | 76 | 2 | 3b |  | 2-11 | UM | Y/N |
| P1921 | Endometrioid | M | 63 | 1 | 1a |  | 2-11 |  | Y/N |
| P1953 | Serous | M | 54 | 3 | 3b |  | 2-11 | UM | Y/Y |
| P1977 | Endometrioid | M | 74 | 1 | 2c |  | 2-11 |  | Y/N |
| P2125 | Serous | M | 56 | 3 | 3c |  | 2-11 |  | Y/Y |
| P2205 | Serous | M | 50 | 3 | 1 |  | 2-11 |  | Y/Y |
| P2281 | Serous | M | 57 | 3 | 3c |  | NS |  | Y/N |
| P2506 | Mixed (Clear Cell*/Serous/ Endometrioid) | M | 75 | 2 | 1 |  | 2-11 |  | Y/N |
| P2712 | Serous | M | - | 3 | 1a |  | 2-11 | UM | Y/Y |
| P2803 | Serous | M | - | 2 | 3c |  | 2-11 |  | Y/Y |
| P2808 | Mucinous | M | 54 | 2 | 3c |  | 2-11 |  | Y/N |
| P4075 | Serous | M | 54 | 2 | 3c |  | 2-11 | UM | Y/Y |
| P4076 | Serous | M | 56 | 3 | 3a |  | 2-11 |  | Y/N |
| P4085 | Serous | M | 79 | 1 | 2c |  | 2-11 | UM | Y/N |
| P4093 | Serous | M | 60 | 3 | 3c |  | 2-11 |  | Y/Y |
| P4178 | Serous | M | - | 3 | 3 |  | 3-11 |  | Y/N |
| P4219 | Serous | M | 44 | 3 | 3c |  | 2-11 |  | Y/Y |
| P4616 | Serous | M | 52 | 3 | 1c |  | 2-11 |  | Y/Y |
| P4684 | Serous | M | 73 | 3 | 3c |  | 2-11 |  | Y/Y |
| P4881 | Clear Cell | M | 34 | 2 | 3 |  | 2-11 |  | Y/N |
| P5390 | Clear Cell | M | 51 | 3 | 3 |  | 2-11 |  | Y/N |
| P5514 | Serous | M | 69 | 3 | 3c |  | NS |  | Y/N |
| P7820 | Serous | M | - | 2 | 2c |  | 2-11 |  | Y/Y |

* Predominant component of mixed tumour; B, Benign; BL, Borderline Malignancy; M, Malignant; HD, Homozygous deletion; NS, Not screened; UM, Unmethylated; Y/Y analysed by both SNP and expression arrays, Y/N, analysed by SNP array only
