# Supplementary material for: Analysis of the Mitogen-activated protein kinase kinase 4 (MAP2K4) tumor suppressor gene in ovarian cancer
Source: BMC Cancer. 2011 May 17;11:173. doi: 10.1186/1471-2407-11-173 (PMC3115913; doi:10.1186/1471-2407-11-173)
Supplement: Additional file 2 — Table S2 Oligonucleotide primer sequences [file 1471-2407-11-173-S2.DOC]

**Supplementary Table 2 Oligonucleotide primer sequences**

| Primer Name | Primer Sequence (5’-3’) | Annealing temp (ºC) | Primer conc (nM) | Buffer^1^ | Length (bp) |
| --- | --- | --- | --- | --- | --- |
| MAP2K4Exon2F | TGTGACTTTCTTATGCCCTCAG | 57 | 300 | MM + M | 225 |
| MAP2K4Exon2R | AAATCTTTGCTTGAGTTGAGCC |
| MAP2K4Exon3F | TGGAAAAATTGCTTCCCAAT | 57 | 300 | MM + M | 277 |
| MAP2K4Exon3R | CAGTGGTATTACTGCACCATGAA |
| MAP2K4Exon4F | CGGTTTTTCTCTACCATGAGAC | 55 | 100 | MM + M | 250 |
| MAP2K4Exon4R | TTAGATATTCATTAGTCAGCTAAAACC |
| MAP2K4Exon5F | CCATTTTAAGTAAAGGCAAGGTG | 57 | 100 | MM + M | 261 |
| MAP2K4Exon5R | TGTGCATTCAAAACTCCAGC |
| MAP2K4Exon6F | ATGCAGAGGACTACACGGGA | 57 | 300 | MM + M | 221 |
| MAP2K4Exon6R | TCAATAACCGTTTCACCAATTA |
| MAP2K4Exon7-1F | TTTTGCTTAAAGTGAAGCCTTATG | 55 | 100 | MM + M | 166 |
| MAP2K4Exon7-1R | GGCAATAGAGTCCACAAGCTG |
| MAP2K4Exon7-2F | TTGCAGATATCAAACCTTCCAA | 55 | 100 | MM | 213 |
| MAP2K4Exon7-2R | TGACTAATGGCCAGCACAAA |
| MAP2K4Exon8F | TGCCTATTCCTTGAGTGTAAGG | 57 | 150 | MM + M | 164 |
| MAP2K4Exon8R | TGTGGCAAGGTTGAATCAG |
| MAP2K4Exon9F | TGATGCCTGGTGTATTTTGC | 55 | 100 | MM + M | 267 |
| MAP2K4Exon9R | CCAATGCTGCTAAGACCAAG |
| MAP2K4Exon10F | TGTGAAAAGAAAAATACTTAGGCAAA | 55 | 100 | MM + M | 162 |
| MAP2K4Exon10R | GAGTTCCGGGGTTAGGATGT |
| MAP2K4Exon11F | TAGCTATGTGTGGTTGGGAGC | 66 | 100 | MM | 338 |
| MAP2K4Exon11R | TTATTGCACATGGTGTCTGGG |
| MAP2K4 Meth F | GGTTTTGTAGTTTAGTATTTGGTT | 50 | 100 | Q | 310 |
| MAP2K4 Meth R | TTCCTTACCCTACATACTACTAAC |
| MAP2K4 cDNA 1F | TCGGTCAACAGTGGATGAAA | 60 | 200 | S | 116 |
| MAP2K4 cDNA 1R | CCTCTCTGAAGAGTGCACCA |
| MAP2K4 cDNA 2F | TCTCCCCGAGTTTCATCAAC | 60 | 200 | S | 122 |
| MAP2K4 cDNA 2R | TCAACGGCACGTTCTTCATA |

1. Buffer MM = Roche High Resolution Melting Master mix with 2.5 mM MgCl_2_; MM + M = Roche High Resolution Melting Master mix with 5 mM MgCl_2_; Q = Qiagen Hotstar *Taq* with 1.5 mM MgCl_2_; S = ThermoScientific SYBR Green mix.
