# Supplementary figures and images for: Analysis of the Mitogen-activated protein kinase kinase 4 (MAP2K4) tumor suppressor gene in ovarian cancer
Source: BMC Cancer. 2011 May 17;11:173. doi: 10.1186/1471-2407-11-173 (PMC3115913; doi:10.1186/1471-2407-11-173)

Supplementary Figure S1

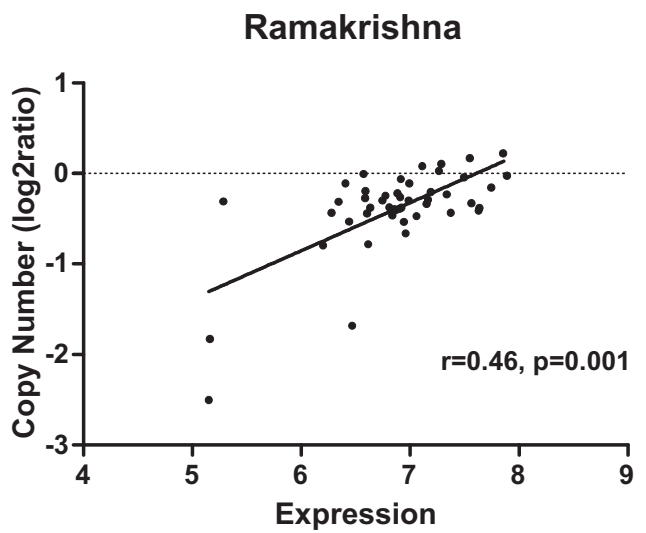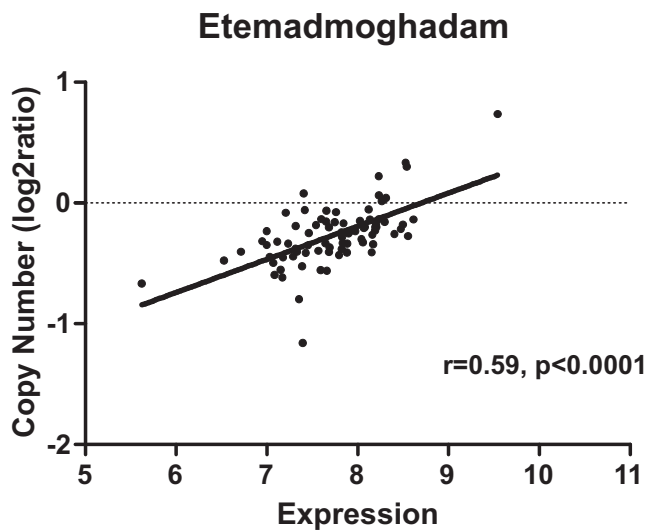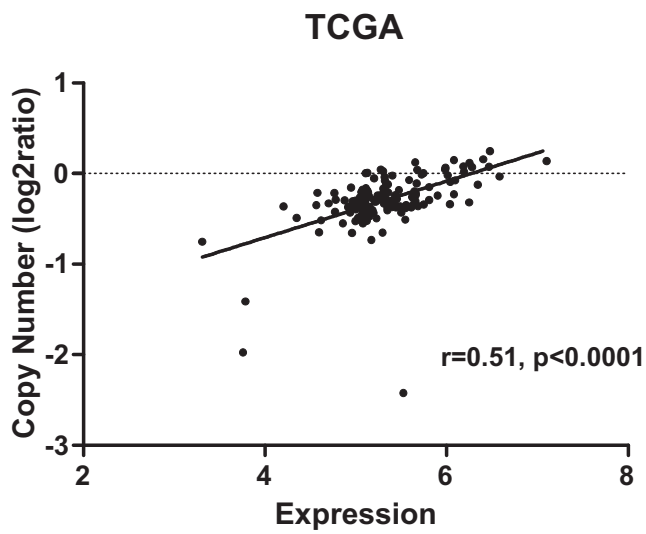

Supplement: Additional file 3 — Figure S1 Correlation of copy number with expression for MAP2K4. Three data sets are shown: Ramakrishna Gene1.0ST/SNP6.0 data [29], TCGA U133A/SNP6.0, and Etemadmoghadam U133Plus2/50K data [30]. For the U133 platforms probeset 203266_s_at is shown. Copy number is taken from the average copy number value of SNPs within the segment of lowest copy number intersecting with the MAP2K4 gene. R and p values for Pearson's correlation. [file 1471-2407-11-173-S3.PDF]

## Supplementary Figure S3

### *MAP2K4* Gene Silencing

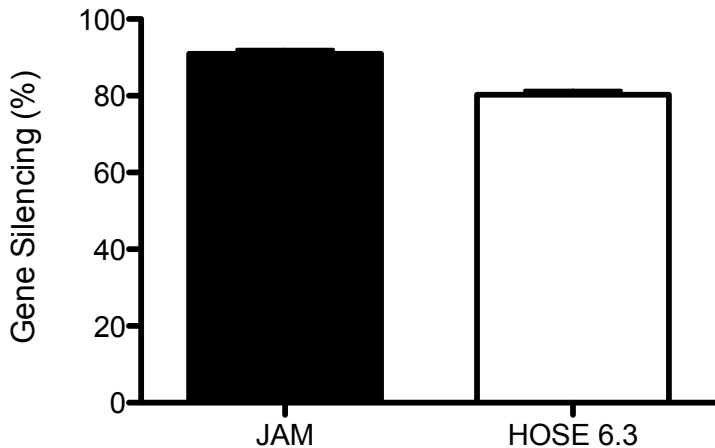

Supplement: Additional file 5 — Figure S3 Reduction in MAP2K4 expression following siRNA knockdown [file 1471-2407-11-173-S5.PDF]
