# Supplementary material for: Analysis of the Mitogen-activated protein kinase kinase 4 (MAP2K4) tumor suppressor gene in ovarian cancer
Source: BMC Cancer. 2011 May 17;11:173. doi: 10.1186/1471-2407-11-173 (PMC3115913; doi:10.1186/1471-2407-11-173)

Supplementary FIgure S2

A

	DF	Estimate	Std Error	W (Wald Chi Square)	p-value (W)	Hazard Ratio
203266_s_at	1	0.539	0.250	4.646	0.031	1.714
Patient Age (Years)	1	0.042	0.012	11.930	0.00055	1.043
0 (Residual Disease)	1	-1.435	0.321	19.936	8.00946e-006	0.238
<1 cm (Residual Disease)	1	-0.150	0.240	0.390	0.532	0.861
>1 cm (Residual Disease)	0	0	0	0	1.000	1.000

B

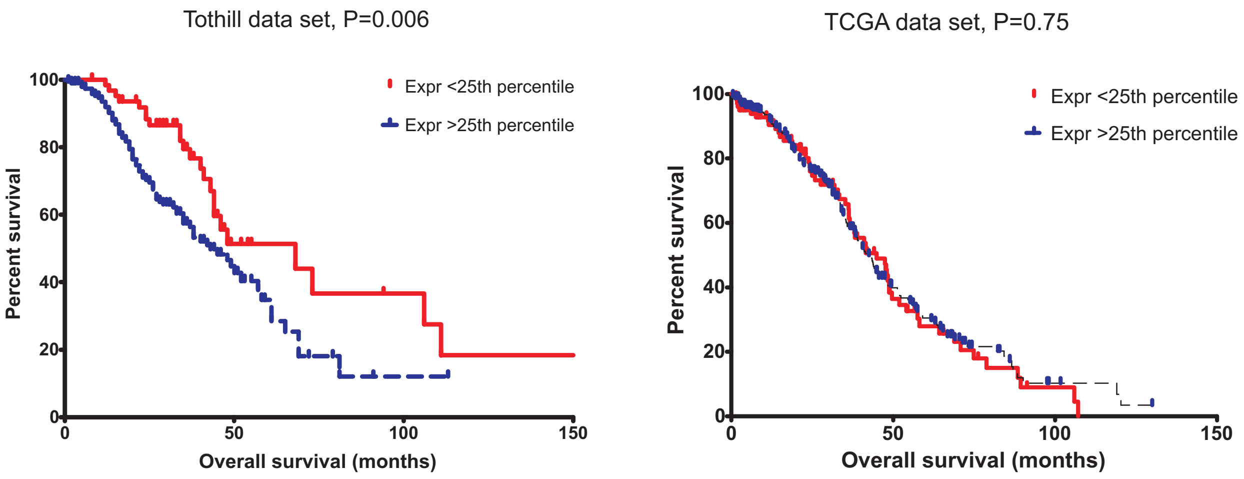

Supplement: Additional file 4 — Figure S2 Overall survival of ovarian cancer patients relative to MAP2K4 expression. A. Partek output for Cox regression analysis in the Tothill data set of MAP2K4 expression probeset 203266_s_at, including patient age and residual disease as co-factors. B. Kaplan-Meier curves, showing difference in survival of patients with low MAP2K4 expression (bottom quartile of cases, red solid line) compared to all remaining cases (blue dashed line) from Tothill et al., 2008 and The Cancer Genome Atlas (TCGA). Graphs truncated at 150 months. P value shown is the log rank (Mantel-Cox) test. [file 1471-2407-11-173-S4.PDF]
